# Graft-recipient-weight ratio and lowered immunosuppression is important for the success of adult liver retransplantation

**DOI:** 10.1038/s41598-023-39007-7

**Published:** 2023-08-07

**Authors:** Jinsoo Rhu, Jieun Kwon, Manuel Lim, Namkee Oh, Sunghyo An, Seung Wook Han, Sung Jun Jo, Sunghae Park, Gyu-Seong Choi, Jong Man Kim, Jae-Won Joh

**Affiliations:** 1 Department of Surgery, Samsung Medical Center, Sungkyunkwan University School of Medicine, 50 Irwon-Dong, Gangnam-Gu, Seoul, 135-710 Korea; 2 Department of Surgery, Soonchunhyang University Seoul Hospital, Soonchunhyang University College of Medicine, Seoul, Korea; 3 Department of Surgery, Myungji Hospital, Hanyang University Medical Center, Goyang, Korea

**Keywords:** Immunology, Gastroenterology

## Abstract

This study analyzed the risk of liver retransplantation and factors related to better outcome. Adult liver transplantations performed during 1996–2021 were included. Comparison between first transplantation and retransplantation were performed. Among retransplantation cases, comparison between whole liver and partial liver graft was performed. Multivariable Cox analyses for analyzing risk factors for primary graft and overall patient survival were performed for the entire cohort as well as the subgroup of patients with retransplantation. A total 2237 transplantations from 2135 adults were included and 103 cases were retransplantation. A total of 44 cases (42.7%) were related to acute graft dysfunction while 59 cases (57.3%) were related to subacute or chronic graft dysfunction. Retransplantation was related poor primary graft (HR 3.439, CI 2.230–5.304, P < 0.001) and overall patient survival. (HR 2.905, CI 2.089–4.040, P < 0.001) Among retransplantations, mean serum FK506 trough level ≥ 9 ng/mL was related to poor primary graft (HR 3.692, CI 1.288–10.587, P = 0.015) and overall patient survival. (HR 2.935, CI 1.195–7.211, P = 0.019) Graft-recipient-weight ratio under 1.0% was related to poor overall patient survival in retransplantations. (HR 3.668, CI 1.150–11.698, P = 0.028). Retransplantation can be complicated with poor graft and patient survival compared to first transplantation, especially when the graft size is relatively small. Lowering the FK506 trough level during the first month can be beneficial for outcome.

## Introduction

Although liver transplantation (LT) can provide excellent cure for end-stage liver disease and malignancies such as hepatocellular carcinoma, there are still patients who require liver retranplantation (reLT) due to acute graft dysfunction, rejection, relapse of the original disease, de novo liver disease or other causes. However, many LT recipients who require reLT fails to receive another liver graft and only certain proportion of recipients with graft failure undergo reLT. The proportion of reLT, therefore, varies around 5–22%, differing by the regions where LT is performed^1–7^. Since reLT is quite rare, published studies were relatively small-sized and only recently, a number of studies using multicenter data were published^3,5^. The data reported showed inferior outcome of reLT compared to first LTs. Reported factors related to outcome were Model for End-stage Liver Disease (MELD) score, donor age, durations between LTs, and intensive care unit admission at the time of LT^3–5^. The study published by Mezochow et al. focused on the intensity of immunosuppression in reLT recipients but failed to distinguish statistically meaningful outcome^5^. The previously published studies mainly had two limitations. Single-center study can be limited by the low number of cases included leading to lower statistical power and weak generalizability. On the other hand, a multicenter study using a national registry can gain statistical power while lack of detailed data can midlead the conclusion by not adjusting for the heterogenous background. In this study, we summarized our single center experience over 25 years, and focused on the risk of reLT compared to first LT, potential risk factors for prognosis in reLT recipients, and our detailed management and its result to find a meaningful information on this rare but devastating patient group.

## Methods

### Decision process for re-transplantation and post-transplantation management

Currently, the Center for Korean Network for Organ Sharing (KONOS) uses the model for end-stage liver disease (MELD) score system which was changed from Child-Turcotte-Pugh score-based status system in 2016. Status 1 comprised fulminant hepatic failure and primary nonfunction within 7 days and Wilson’s disease with fulminant hepatic failure. For primary LT, deceased donor LT is considered for patients who have high MELD score. For fulminant hepatic failure, the patient condition is considered for deciding whether to transplant deceased donor’s liver or living donor’s liver. When the severity of encephalopathy is severe, living donor LT can be considered to prevent brain damage. However, deceased donor can be allocated during the preparation process. Therefore, this is basically, case-by-case strategy. When there is patient with chronic liver disease with low MELD score beneath 35, living donor LT is considered since it is difficult to be allocated in Korea. Hepatocellular carcinoma patients are reviewed for the availability of living donor. When a recipient with primary or secondary graft dysfunction, deceased donor LT is performed since there is a high probability to be allocated. However, when the liver failure process is slow, living donor LT is considered if there is a potential living donor who is willing to donate.

The standard regimen for immunosuppression comprises induction therapy and maintenance regimen. Induction therapy includes basiliximab and methylprednisolone. Maintenance regimen includes tacrolimus, mycophenolate mofetil and oral methylprednisolone. The tacrolimus trough level is maintained around 8–10 ng/mL during the first month, and gradually tapered down. For re-transplantation, induction therapy varies among patients. Sometimes basiliximab is not given. Maintenance regimen can be delayed or maintained with lower level than standard regimen. However, this is based on individual patient’s condition.

### Patients and data

LTs for adult recipients performed from 1996 to October 2021 at Samsung Medical Center were included. Pediatric LTs were excluded from the study. Donor and recipient data were comprehensively reviewed from our prospectively maintained database especially focusing on reLT patients. Recipient’s complications that occurred within 30 days post-LT were collected and categorized according to Clavien–Dindo classification^8^. Data on graft survival and overall survival were collected. Primary graft failure was defined as occurrence of graft failure without any other secondary cause. Overall graft failure was defined as both primary and secondary graft failure. Therefore, overall graft failure includes graft failure occurring as the process of multiorgan failure of various causes.

#### Patients with retransplantation

Reason for reLT as well as cause of death were reviewed in detail. The reasons for reLT were categorized into acute graft dysfunction and subacute or chronic graft dysfunction. To categorize cases into acute and subacute or chronic, days after the prior LT were calculated and medical charts were reviewed for detailed history. Acute graft dysfunction included primary nonfunction and secondary graft dysfunction which showed failure of engraftment after prior LT. On the other hand, subacute or chronic graft dysfunction were defined as cases in which the graft showed graft failure after successful engraftment which can be judged by normalizing liver chemistries after prior LT. Whether the recipients were under ventilator care or infusion of inotropic agents for management of shock.

To review how the reLT recipients were managed by the medical team, use of immunosuppressants were also collected. Use of induction therapy and maintenance regimen during the first month after reLT were collected. The initiation day after reLT as well as the mean trough level of tacrolimus after reLT were calculated. The initiation day after reLT of mycophenolate mofetil and the median daily dosage were calculated. The medican daily dosage of steroid were also calculated.

Infection episodes occurring during the first month after reLT were reviewed. Respiratory infections were subdivided into bacterial pneumonia, aspergillus pneumonia and viral pneumonia. Intra-abdominal infection was defined as culture-positive drain with signs of infection based on blood chemistry and computed tomography of the abdomen. Although cytomegalovirus (CMV) antigenemia was not included in infection episode, positivity of the test as well as CMV count among 200,000 copies were reviewed.

### Statistical analysis

For the entire patient cohort, statistical analyses were performed focusing on the prognosis of reLT compared to first LT. Comparison of baseline characteristics as well as outcome between first LT and reLT were performed. Comparisons were performed based on cases performed, but not based on patients. Furthermore, comparison between reLT patients with partial liver graft and whole liver graft was performed.

Kaplan–meier survival analyses with log-rank test were performed to analyze primary graft survival, overall graft survival and overall patient survival. Multivariable Cox analyses were performed to analyze potential risk factors for primary graft survival and overall survival. In the subgroup analysis including reLT patients, graft-recipient-weight ratio (GRWR) was divided into two groups with a cutoff of 1% while first month mean serum tacrolimus trough level was divided with a cutoff of 9 ng/mL where it showed the lowest P-value.

Numerical variables of each group were compared using Student’s t-test or Mann–Whitney test and expressed as mean ± standard deviation or median (interquartile range, IQR), respectively. Categorical variables divided into two components were compared using chi-square test or Fisher’s exact test. Statistical analysis was executed using Statistical Package for Social Sciences (SPSS) version 22.0 (IBM Corp., Armonk, NY, USA). This study was approved by the Institutional Review Board of Samsung Medical Center (IRB No. 2022-03-097) and the study was performed in accordance with relevant guidelines and regulations. This study did not include human participants and informed consent was waived by the institutional review board of Samsung Medical Center.

## Results

During the study period, a total of 2392 recipients underwent 2513 LTs at Samsung Medical Center. After excluding 258 pediatric recipients who underwent 276 LTs, 2237 LTs of 2135 adult recipients were included to the study. Among these cases, 2134 first LTs were performed by 2134 recipients while one patient who underwent first LT at a different institution received retransplantation at Samsung Medical Center. Among 2134 recipients, 99 recipients received second transplantation and four of them underwent third LT afterwards. The reasons for reLT were acute graft dysfunction in 44 cases (42.7%), subacute or chronic graft dysfunction in 55 cases (53.4%) and recurrence of hepatocellular carcinoma in 3 cases. (2.9%) Among acute graft dysfunction, primary nonfunction (n = 22) accounted for 21.4% while secondary graft dysfunction (n = 22) accounted for 21.4%. The reasons for secondary graft dysfunction were hepatic artery complications (n = 11), portal vein complications (n = 6), hepatic vein complications (n = 2), massive bleedings (n = 3) and bile duct complication (n = 1). The reasons for subacute or chronic graft dysfunction were biliary complications (n = 19), chronic rejection (n = 26), ischemic damage of graft (n = 4), hepatitis B recurrence (n = 4) and alcoholic cirrhosis (n = 2).

### Comparison between first LT and reLT

Table 1 summarizes the comparison between first LT group (n = 2134) and reLT group (n = 103). In general, the two groups showed significant differences in recipient-, donor-, and also outcome-related variables. Proportion of hepatitis B virus was prominent in first LT group (66.7% vs. 54.4%, P = 0.010). Hepatocellular carcinoma rate was also higher in the first LT group (52.3% vs. 36.3%, P = 0.002). Regarding pre-transplant chemistries, the reLT group showed more disease profile including serum albumin (P = 0.008), serum total bilirubin (P < 0.001), prothrombin time (P = 0.002) and serum creatinine (P = 0.001). Pre-transplant variceal bleeding showed higher rate in the first LT group (16.3% vs. 4.9%, P = 0.001) as well as ascites (59.5% vs. 42.7%, P = 0.001). However, hepatorenal syndrome rate was higher in the reLT group (12.7% vs. 48.5%, P < 0.001) as well as pre-transplant ventilator care (4.4% vs. 21.4%, P < 0.001) mean MELD score (20.3 ± 11.6 vs. 32.1 ± 8.8, P < 0.001) and Child-Turcot-Pugh (CTP) score was higher in the reLT group. (9.1 ± 2.7 vs. 10.3 ± 2.0, P < 0.001).

**Table 1:** Comparison of baseline characteristics and transplantation-related outcome of liver transplantation recipients between first transplantation group and retransplantation group.

		First transplantation (n = 2134)	Retransplantation (n = 103)	P-value
Recipient-related variable	Recipient sex (male vs. female)	1577/557 (73.9%)	71/32 (68.9%)	0.232
	Recipient age (years)	47.2 ± 17.7	44.0 ± 18.6	0.060
	Hypertension	274 (12.8%)	8 (7.8%)	0.130
	Diabetes mellitus	458 (21.5%)	27 (26.2%)	0.253
	Etiology (Hepatitis B virus vs. others)			0.010
	Hepatitis B virus	1414 (66.7%)	56 (54.4%)	
	Hepatitis C virus	97 (4.6%)	5 (4.9%)	
	Alcohol	321 (15.1%)	12 (11.7%)	
	Others	289 (13.6%)	30 (29.1%)	
	Hepatocellular carcinoma	1108 (52.3%)	37 (36.3%)	0.002
	Pre-transplant albumin	3.2 ± 0.9	3.1 ± 0.4	0.008
	Pre-transplant total bilirubin	10.9 ± 14.0	22.3 ± 15.2	< 0.001
	Pre-transplant prothrombin time, INR	2.05 ± 2.00	2.51 ± 1.55	0.002
	Pre-transplant creatinine	1.3 ± 1.2	2.1 ± 1.8	0.001
	Hepatic encephalopathy	501 (23.5%)	22 (21.4%)	0.620
	Variceal bleeding	347 (16.3%)	5 (4.9%)	0.001
	Ascites	1269 (59.5%)	44 (42.7%)	0.001
	Hepatorenal syndrome	271 (12.7%)	50 (48.5%)	< 0.001
	Spontaneous bacterial peritonitis	204 (9.6%)	6 (5.8%)	0.204
	Pretransplant ventilator care	94 (4.4%)	22 (21.4%)	< 0.001
	MELD score	20.3 ± 11.6	32.1 ± 8.8	< 0.001
	CTP score	9.1 ± 2.7	10.3 ± 2.0	< 0.001
Donor-related variable	Type of transplantation			< 0.001
	Living donor	1636 (76.7%)	12 (11.7%)	
	Deceased donor	498 (23.3%)	91 (88.3%)	
	Donor sex (male vs. female)	1374/760 (64.4%)	64/39 (62.1%)	0.642
	Donor age (years)	36.0 ± 14.1	46.0 ± 15.2	< 0.001
	ABO incompatibility	224 (10.5%)	2 (1.9%)	0.002
	Graft weight	849 ± 343	1316 ± 375	< 0.001
	Graft-recipient weight ratio	1.28 ± 0.55	2.11 ± 0.67	< 0.001
	Warm ischemic time	40.2 ± 26.2	35.0 ± 15.1	0.066
	Cold ischemic time	134 ± 114	287 ± 158	< 0.001
	Graft type (whole vs. partial)			< 0.001
	Whole liver	475 (22.5%)	88 (86.3%)	
	Right liver	1595 (75.7%)	14 (13.7%)	
	Left liver	36 (1.7%)	–	
	Right posterior	1 (0.0%)	–	
Recipient outcome	30-day complication	1079 (50.6%)	82 (80.6%)	< 0.001
	None	1050 (49.2%)	20 (19.4%)	< 0.001
	Grade I/II	315 (14.8%)	14 (13.6%)	
	Grade III	610 (28.6%)	35 (34.0%)	
	Grade IV/V	159 (7.5%)	34 (33.0%)	
	Primary graft failure	184 (8.6%)	34 (33.0%)	< 0.001
	Secondary graft failure	596 (27.9%)	60 (58.3%)	< 0.001
	Death	541 (25.6%)	55 (53.4%)	< 0.001

*MELD* model for end-stage liver disease, *CTP* Child-Turcot-Pugh.

Majority of reLT recipients received their grafts from deceased donor (88.3%) while only 23.3% of cases were performed as deceased donor LT in the first LT group (P < 0.001). Donor age was significantly higher in the reLT group (46.0 ± 15.2 years) compared to the first LT group (36.0 ± 14.1 years, P < 0.001). While ABO incompatible LTs comprised 10.5% of the first LT group, only 1.9% of cases were ABO incompatible in the reLT group (P = 0.002). Mean graft weight (849 ± 343 g vs. 1316 ± 375 g, P < 0.001) and GRWR (1.28 ± 0.55% vs. 2.11 ± 0.67%, P < 0.001) were higher in the reLT group. Whole liver graft transplantation was prominently performed in the reLT group (86.3%) compared to the first LT group (22.5%, P < 0.001).

Patient outcomes were poorer in the reLT group, including 30-day complication rate (50.6% vs. 80.6%, P < 0.001) as well as the severity classified by Clavien–Dindo classification (P < 0.001). Primary graft failure rate (8.6% vs. 33.0%, P < 0.001), overall graft failure rate (27.9% vs. 58.3%, P < 0.001) and mortality rate (25.6% vs. 53.4%, P < 0.001) were both higher in the reLT group.

### Primary graft survival, overall graft survival and overall patient survival

**Figure 1:**
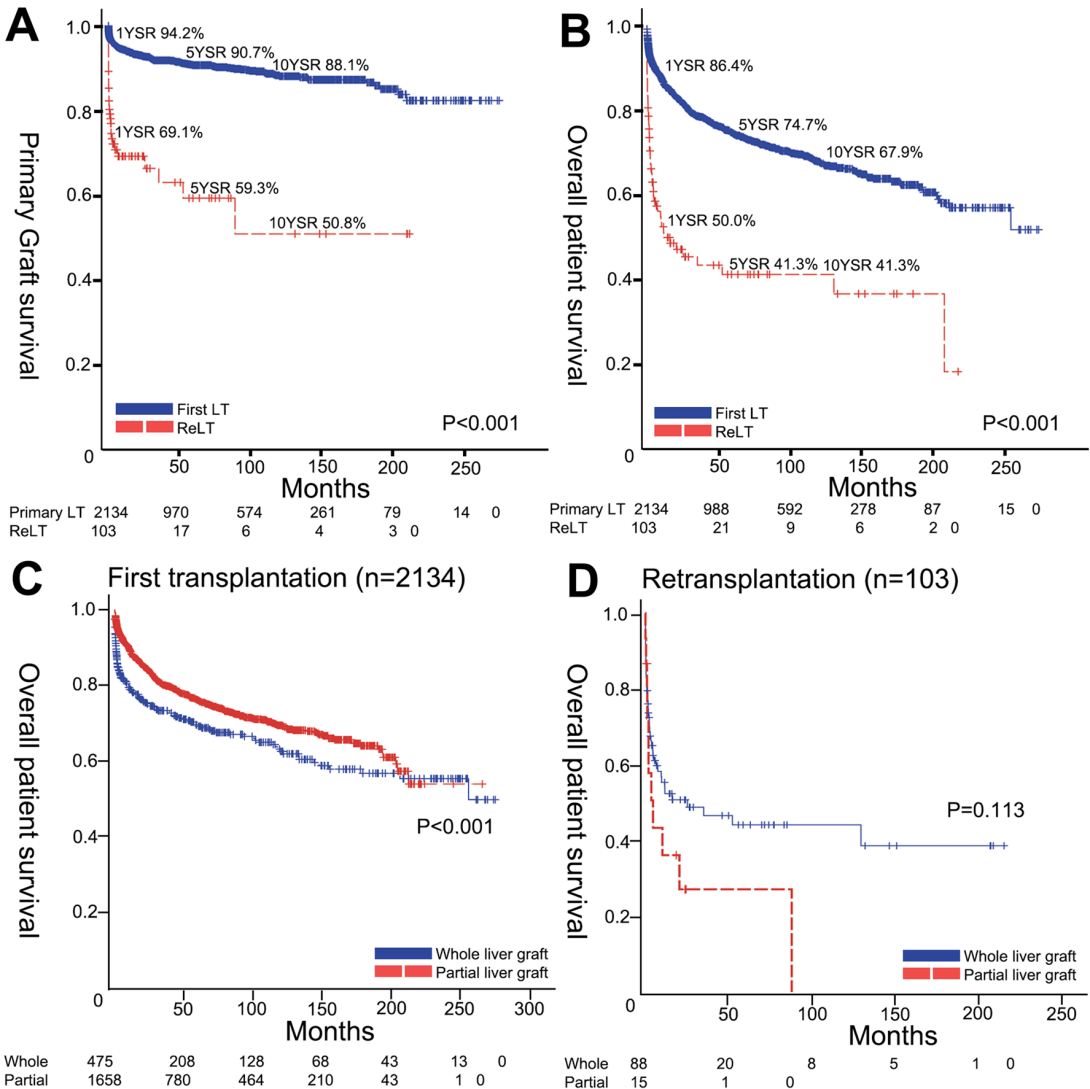
Kaplan–Meier survival curves of patients included. (**A**) Primary graft survivals and (**B**) overall patient survivals according to the first transplantation and retransplantation. (**C**) Overall patient survivals according to whole liver graft and partial liver graft in patients with first transplantation. (**D**) Overall patient survival curves according to whole liver graft and partial liver graft in patients with retransplantation.

Kaplan–Meier survival analyses with log-rank tests were performed to compare primary graft survival, overall graft survival and overall patient survival of first LT group and reLT group as well as the difference between types of liver grafts in the subgroups with first LT group and the reLT group (Fig. 1). The 1-year, 5-year, and 10-year primary graft survivals of the first LT group and the reLT group were 94.2%, 90.7%, 88.1% and 69.1%, 59.3%, 50.8%, respectively (P < 0.001). The 1-year, 5-year, and 10-year overall graft survivals of the first LT group and the reLT group were 84.9%, 72.3%, 64.9% and 45.3%, 36.5%, 31.2%, respectively (P < 0.001). The 1-year, 5-year, and 10-year overall patient survivals of the first LT group and the reLT group were 86.4%, 74.7%, 67.9% and 50.0%, 41.3%, 41.3%, respectively (P < 0.001). To evaluate the impact of partial liver and whole liver graft, overall survivals were analyzed in two separate subgroups. In patients with first LT, partial liver graft showed better overall survival compared to the whole liver graft (P < 0.001). On the other hand, in patients with reLT, partial liver graft showed inferior survival curve without statistical significance (P = 0.113).

Multivariable Cox analyses were performed to identify significant risk factors for prognosis (Table 2). ReLT (HR 3.439, CI 2.230–5.304, P < 0.001) was a significant risk factor for poor primary graft survival along with MELD score (HR 1.020, CI 1.007–1.033, P = 0.002) and donor age (HR 1.021, CI 1.011–1.032, P < 0.001). ReLT (HR 2.905, CI 2.089–4.040, P < 0.001) showed significant relationship to overall survival along with recipient age ≥ 60 years (HR 1.473, CI 1.207–1.797, P < 0.001), hepatorenal syndrome (HR 1.437, CI 1.083–1.907, P = 0.012), and pretransplant albumin (HR 1.227, CI 1.147–1.314, P < 0.001) were significant factor related to overall survival. Factors related to overall graft survival were reLT (HR 3.026, CI 2.233–4.101, P < 0.001), MELD score (HR 1.014, CI 1.007–1.021, P < 0.001), recipient age ≥ 60 (HR 1.294, CI 1.066–1.570, P = 0.009), pretransplant albumin (HR 1.191, CI 1.116–1.270, P < 0.001), and donor age (HR 1.011, CI 1.005–1.017, P < 0.001, Supplementary Table 1).

**Table 2:** Multivariable Cox analyses of potential risk factors for primary graft survival and overall patient survival of patients who underwent liver transplantation.

Variables	n	Primary graft survival						Overall patient survival					
		Univariable			Multivariable (final model)			Univariable			Multivariable (final model)		
		HR	CI	P	HR	CI	P	HR	CI	P	HR	CI	P
Retransplantation	103	5.882	4.048–8.545	< 0.001	3.439	2.230–5.304	< 0.001	3.459	2.618–4.570	< 0.001	2.905	2.089–4.040	< 0.001
Partial graft (against whole)	1659	0.407	0.310–0.533	< 0.001				0.634	0.533–0.753	< 0.001	0.339	0.084–1.372	0.129
MELD score		1.035	1.024–1.046	< 0.001	1.020	1.007–1.033	0.002	1.017	1.010–1.024	< 0.001	1.008	0.999–1.017	0.071
Male recipient (vs. female)	1635	0.770	0.576–1.030	0.078				1.038	0.859–1.253	0.702			
Recipient age ≥ 60	509	1.029	0.738–1.435	0.867				1.424	1.180–1.718	< 0.001	1.473	1.207–1.797	< 0.001
Hepatocellular carcinoma	1139	0.674	0.514–0.884	0.004				1.129	0.960–1.328	0.141			
Living donor (vs. deceased donor)	1633	0.417	0.319–0.547	< 0.001				0.635	0.535–0.754	< 0.001	2.812	0.692–11.426	0.148
Hypertension	277	0.951	0.617–1.467	0.821				1.219	0.955–1.556	0.113			
Diabetes mellitus	481	1.187	0.867–1.624	0.286				1.160	0.958–1.406	0.129			
Hepatic encephalopathy	522	1.158	0.860–1.560	0.334				1.060	0.884–1.272	0.528			
Variceal bleeding	352	0.527	0.344–0.807	0.003	0.684	0.440–1.064	0.092	0.771	0.620–0.960	0.020			
Ascites	1304	0.973	0.738–1.284	0.848				0.818	0.693–0.965	0.017			
Hepatorenal syndrome	316	2.826	2.070–3.858	< 0.001				2.111	1.711–2.606	< 0.001	1.437	1.083–1.907	0.012
Spontaneous bacterial peritonitis	210	0.809	0.514–1.273	0.360				1.059	0.833–1.347	0.639			
Pretransplant ventilator care	116	2.843	1.858–4.349	< 0.001				1.652	1.188–2.295	0.003			
Pretransplant albumin		0.979	0.791–1.211	0.844				1.190	1.109–1.276	< 0.001	1.227	1.147–1.314	< 0.001
ABO incompatible	225	1.413	0.928–2.151	0.107				0.802	0.579–1.112	0.186			
Male donor (vs. female)	1427	0.854	0.649–1.122	0.257				0.899	0.760–1.062	0.209			
Donor age		1.028	1.021–1.035	< 0.001	1.021	1.011–1.032	< 0.001	1.013	1.008–1.019	< 0.001	1.006	1.000–1.013	0.066
Warm ischemic time		1.001	0.995–1.007	0.749				1.000	0.997–1.004	0.856			
Cold ischemic time		1.003	1.002–1.003	< 0.001	1.001	1.000–1.002	0.068	1.001	1.001–1.002	< 0.001			
GRWR		1.789	1.459–2.194	< 0.001				1.436	1.252–1.647	< 0.001			
GRWR < 1.0	672	0.656	0.477–0.902	0.009				0.758	0.630–0.912	0.003			

*LT* Liver transplantation, *reLT* Liver retransplantation, *RRT* Renal replacement therapy, *MELD* model for end-stage liver disease, *GRWR* Graft-recipient weight ratio.

### Comparison between reLT patients with whole liver graft and whole liver graft

Table 3 summarizes the comparison of background characteristics between reLT patients with whole liver graft and partial liver graft. Among 103 reLT cases, whole liver was transplanted in 88 cases (85.4%) while partial liver was transplanted in 15 cases. (14.6%) While there were no difference in the type of donor for previous transplant (27.3% living donor in whole liver group vs. 26.7% living donor in partial liver group, P = 1.000), significant difference was present in donor type during reLT (100.0% deceased donor in whole liver group vs. 20.0% deceased donor in partial liver group, P < 0.001). Hospitalization prior to reLT was significantly different between the two groups. (93.2% in whole liver group vs. 60.0% in partial liver group, P < 0.001) Median graft weight (1418 g, IQR 1204–1604 in whole liver group vs. 718 g, IQR 614–958 in partial liver group, P < 0.001) as well as median GRWR (2.22%, IQR 1.90–2.66 in whole liver group vs. 1.10%, IQR 0.92–1.66 in partial liver group, P < 0.001) were significantly different between the two groups.

**Table 3:** Comparisons of background characteristics between retransplantation patients according to their graft type.

	Retransplantation with whole liver (n = 88)	Retransplantation with partial liver (n = 15)	P-value
Recipient sex (male vs. female)	58/30 (65.9%)	13/2 (86.7%)	0.138
Recipient age (years)	52 (44–57)	53 (47–59)	0.443
Previous transplant			1.000
Living donor	24 (27.3%)	4 (26.7%)	
Deceased donor	64 (72.7%)	11 (73.3%)	
Retransplant			< 0.001
Living donor	–	12 (80.0%)	
Deceased donor	88 (100.0%)	3 (20.0%)	
Etiology (Hepatitis B virus vs. others)			0.931
Hepatitis B virus	48 (54.5%)	8 (53.3%)	
Hepatitis C virus	2 (2.3%)	3 (20.0%)	
Alcohol	12 (13.6%)	–	
Others	26 (29.5%)	4 (26.7%)	
Reason for re-transplantation (acute vs. other)			0.427
Acute graft dysfunction	39 (44.3%)	6 (40.0%)	
Primary nonfunction	19 (21.6%)	3 (20.0%)	
Secondary graft nonfunction	20 (22.7%)	2 (13.3%)	
Hepatic artery complication	9 (10.2%)	1 (6.7%)	
Portal vein complication	5 (5.7%)	1 (6.7%)	
Hepatic vein complication	2 (2.3%)	–	
Massive bleeding	3 (3.4%)	–	
Bile duct complication	1 (1.1%)	–	
Subacute or chronic graft dysfunction	49 (55.7%)	6 (40.0%)	
Biliary complication	18 (20.5%)	1 (6.7%)	
Chronic rejection	23 (26.1%)	3 (20.0%)	
Ischemic damage of graft	3 (3.4%)	1 (6.7%)	
HBV recurrence	3 (3.4%)	1 (6.7%)	
Alcoholic cirrhosis	2 (2.3%)	–	
HCC recurrence	–	3 (20.0%)	
Median days after previous transplantation (IQR)	218 (9.25–1151)	337 (22–1748)	0.352
Median days between registration for deceased donor and re-transplantation (days)	9 (3–67.5)	54.5 (7.25–229.5)	0.059
Hepatocellular carcinoma	31 (35.6%)	6 (40.0%)	0.745
Pre-transplant albumin	3.0 (2.8–3.3)	3.2 (2.9–3.4)	0.095
Pre-transplant total bilirubin	21.0 (10.1–35.9)	18.1 (5.7–37.7)	0.575
Pre-transplant prothrombin time, INR	2.10 (1.48–3.03)	1.70 (1.14–2.31)	0.073
Pre-transplant creatinine	1.7 (1.0–2.9)	1.4 (0.7–3.1)	0.379
MELD score	34 (28–40)	29 (14–39)	0.123
Hepatic encephalopathy	20 (22.7%)	2 (13.3%)	0.516
Variceal bleeding	3 (3.4%)	2 (13.3%)	0.153
Ascites	37 (42.0%)	7 (46.7%)	0.738
Hepatorenal syndrome	44 (50.0%)	6 (40.0%)	0.474
Spontaneous bacterial peritonitis	6 (6.8%)	–	0.589
Ventilator care	19 (21.6%)	3 (20.0%)	1.000
Pretransplant inotropics	27 (31.8%)	4 (26.7%)	0.772
Pretransplant hospitalization	82 (93.2%)	9 (60.0%)	< 0.001
Donor sex (male vs. female)	54/34 (61.4%)	10/5 (66.7%)	0.696
Donor age	49 (37–59)	53 (32–55)	0.694
Graft weight	1418 (1204–1604)	718 (614–958)	< 0.001
Graft-recipient weight ratio	2.22 (1.90–2.66)	1.10 (0.92–1.66)	< 0.001
Warm ischemic time	32 (26–41)	34 (29–46)	0.347
Cold ischemic time	295 (247–395)	86 (59–152)	< 0.001

Table 4 summarizes the comparisons regarding posttransplant management and transplantation outcome between the two groups. There were no significant difference between the two groups regarding proportion of induction therapy as well as the intensity (P = 0.555 and P = 0.425). There was a trend toward significance regarding mean trough level of serum tacrolimus during 1st month. Median mean FK trough level was 6.7 ng/mL (IQR 5.5–11.1) in the partial liver group while that of whole liver group was 5.7 ng/mL (IQR 4.2–7.1, P = 0.068).

**Table 4:** Comparisons of post-transplantation management and outcome between retransplantion patients according to their graft types.

	Retransplantation with whole liver (n = 88)	Retransplantation with partial liver (n = 15)	P-value
Basiliximab induction	48 (58.5%)	10 (71.4%)	0.555
Induction therapy other than steroid			0.425
None	17 (20.2%)	3 (21.4%)	
None due to recent induction with previous LT	17 (20.2%)	1 (7.1%)	
Single dose of 20 mg basiliximab	13 (15.5%)	1 (7.1%)	
40 mg of basiliximab divided by two doses	35 (41.7%)	9 (64.3%)	
Antithymocyte globulin	2 (2.4%)	–	
Day of tacrolimus initiation, median	5 (3–6)	4 (3–5)	0.122
Mean trough level of tacrolimus during 1 month (ng/dL)	5.7 (4.2–7.1)	6.7 (5.5–11.1)	0.068
Day of mycophenolate mofetil initiation, median	2 (1–4)	1 (1–3)	0.223
Median daily dosage of mycophenolate mofetil (mg)	411 (111–763)	406 (83–687)	0.805
Median daily dosage of methylprednisolone (mg)	56 (50–74)	67 (55–74)	0.288
Infection of any type within 1 month	42 (52.5%)	8 (57.1%)	0.748
Pneumonia within 1 month			0.461
Bacterial pneumonia	8 (10.0%)	3 (21.4%)	0.360
Aspergillus pneumonia	6 (7.5%)	1 (7.1%)	0.340
Viral pneumonia	1 (1.2%)	–	1.000
Intra-abdominal infection within 1 month	20 (25.0%)	4 (28.6%)	0.749
CMV antigenemia within 1 month	53 (64.6%)	10 (71.4%)	0.765
CMV count, median (/200,000)	7 (0–41)	14 (0–46)	
Blood borne infection			0.728
Bacteremia	11 (13.8%)	2 (14.3%)	
Candidemia	3 (3.8%)	–	
Viremia other than CMV	1 (1.2%)	1 (7.1%)	
Recipient 30-day complication	71 (80.7%)	12 (80.0%)	1.000
Clavien–Dindo classification			
None	17 (19.3%)	3 (20.0%)	0.982
Grade I/II	12 (13.6%)	2 (13.3%)	
Grade III	30 (34.1%)	5 (33.3%)	
Grade IV/V	29 (33.0%)	5 (33.3%)	
Rejection	19 (21.6%)	2 (13.3%)	0.730
T cell mediated rejection	18 (20.5%)	2 (13.3%)	
Antibody mediated rejection	1 (1.1%)	–	
Graft-versus-host disease	2 (2.3%)	–	
Graft failure (n)	30 (34.1%)	4 (26.7%)	0.768
Median duration from retransplantation to graft failure (months)	0.23 (0.03–2.00)	1.25 (0.16–67.15)	0.485
Death	44 (50.0%)	11 (73.3%)	0.160
Median duration from retransplantation to death (months)	1.72 (0.27–6.12)	1.91 (0.95–10.35)	0.412
Median duration of follow up	6.21 (1.43–33.82)	3.65 (0.95–20.70)	0.413
Death due to acute graft dysfunction			1.000
Primary nonfunction	9 (20.5%)	2 (18.2%)	
Secondary graft dysfunction	5 (11.4%)	–	
Other causes of death			0.056
Chronic graft rejection	3 (6.8%)	1 (9.1%)	
Acute graft rejection	2 (4.5%)	–	
Hypovolemic shock	4 (9.1%)	2 (18.2%)	
Infection, pulmonary	6 (13.6%)	1 (9.1%)	
Infection, intra-abdominal	6 (13.6%)	3 (27.3%)	
Graft-versus-host disease	2 (4.5%)	–	
Progression of hepatocellular carcinoma	2 (4.5%)	1 (9.1%)	
De novo malignancy	1 (2.3%)	–	
Neurologic accident	4 (9.1%)	–	
Accidental outside hospital	–	1 (9.1%)	

### Primary graft survival and overall patient survival in reLT patients

**Figure 2:**
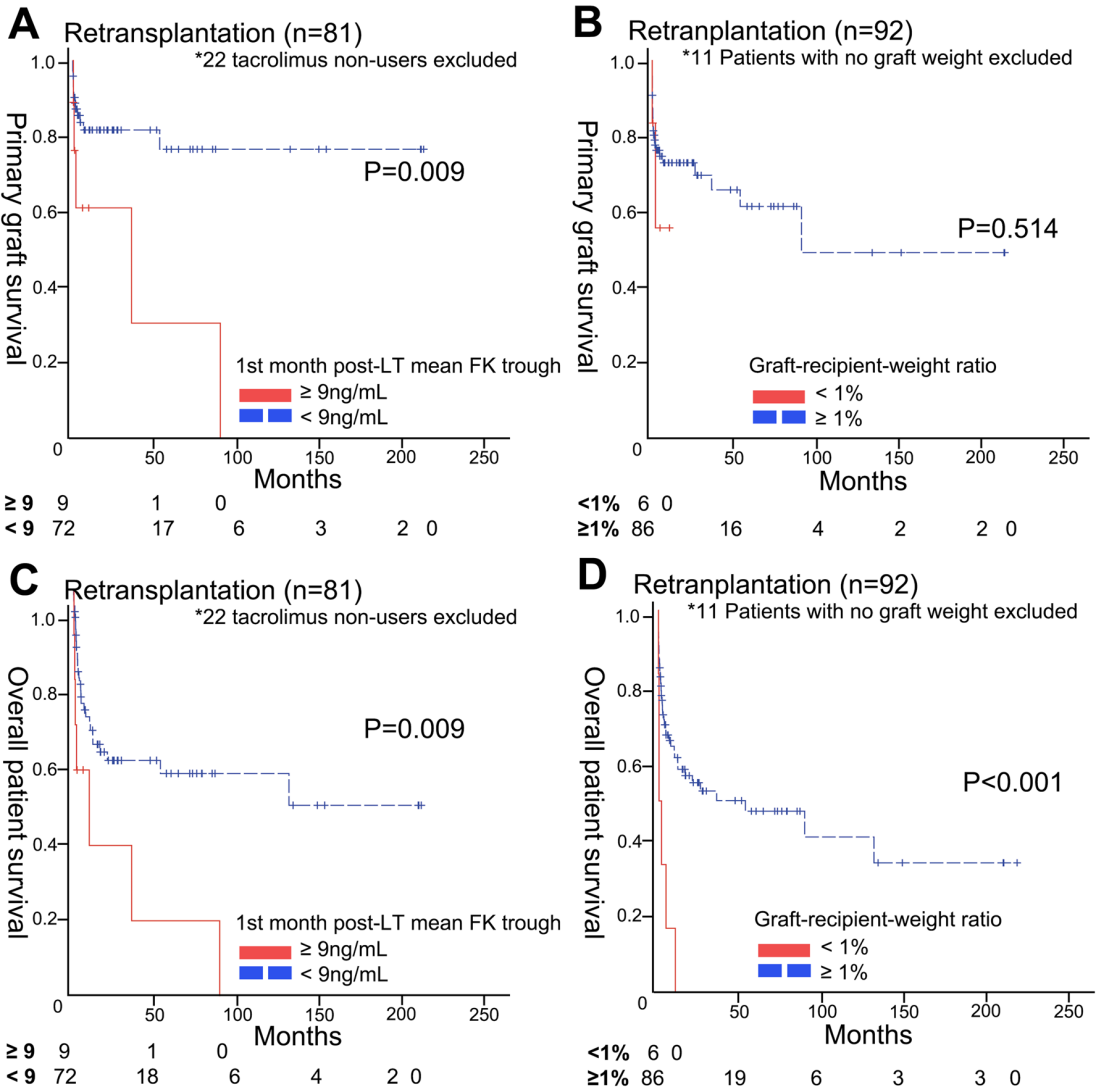
Kaplan–Meier survival curves of retransplantation cases. (**A**) Primary graft survivals of retransplantation cases according to the first month post-LT mean FK trough level (**B**) and graft-recipient-weight ratio. (**C**) Overall patient survivals of retransplantation cases according to the first month post-LT mean FK trough level (**D**) and graft-recipient-weight ratio.

Primary graft survivals and overall survivals were analyzed using Kaplan–meier log-rank test according to GRWR < 1.0% and mean daily FK trough level ≥ 9 ng/mL in reLT patients (Fig. 2). Based on log-rank test, graft survival (P = 0.009) and overall survival (P = 0.009) showed significant difference according to the mean daily FK trough level of ≥ 9 ng/mL. While GRWR < 1% was not significantly related to graft survival difference (P = 0.514), it showed significant difference in overall survival (P < 0.001).

Multivariable Cox analysis of potential risk factors for primary graft survival and overall patient survival in reLT patients were analyzed (Table 5). In the univariable analyses for primary graft survival, only mean daily FK trough level ≥ 9 ng/mL (HR 3.692, CI 1.288–10.587, P = 0.015) was related to poor graft survival. Regarding overall patient survival, GRWR < 1.0% (HR 3.668, CI 1.150–11.698, P = 0.028) and mean daily FK trough level ≥ 9 ng/mL (HR 2.935, CI 1.195–7.211, P = 0.019) were significantly related to poor overall survival. When variables with post-transplant immunosuppression is excluded, GRWR < 1% was the only factor related to poor survival (HR 4.065, CI 1.676–9.861, P = 0.002). Supplementary Table 2 shows the multivariable analysis for overall graft survival. GRWR < 1.0% (HR 3.029, CI 1.018–9.018, P = 0.046) and mean daily FK trough level ≥ 9 ng/mL (HR 2.716, CI 1.201–6.141, P = 0.016) were significantly related to poor overall graft survival.

**Table 5:** Multivariable Cox analysis of potential risk factors for primary graft survival and overall patient survival in patients with retransplantation.

Variables	n	Primary graft survival			Overall patient survival								
		Univariable			Univariable			Multivariable (FK included)			Multivariable (FK excluded)		
		HR	CI	P	HR	CI	P	HR	CI	P	HR	CI	P
GRWR		0.894	0.507–1.578	0.700	0.650	0.415–1.018	0.060						
GRWR < 1.0%	6	1.607	0.377–6.855	0.521	4.243	1.758–10.241	0.001	3.668	1.150–11.698	0.028	4.065	1.676–9.861	0.002
Living donor for first transplant	75	0.994	0.446–2.218	0.989	1.016	0.552–1.871	0.958						
Living donor for retransplant	12	1.068	0.375–3.038	0.902	1.748	0.853–3.583	0.127						
Acute dysfunction (against chronic)	44	1.204	0.613–2.367	0.589	0.956	0.556–1.643	0.870						
Days between transplantations ≥ 30 days	70	1.208	0.577–2.530	0.616	1.456	0.793–2.674	0.226						
Partial graft (against whole)	15	0.814	0.286–2.318	0.700	1.700	0.874–3.305	0.118						
MELD score		1.006	0.967–1.047	0.767	1.019	0.986–1.053	0.258						
Male recipient (vs. female)	71	0.712	0.349–1.453	0.351	1.246	0.686–2.264	0.470						
Recipient age ≥ 60 years	19	0.737	0.284–1.916	0.532	1.400	0.735–2.668	0.306						
Hepatocellular carcinoma	37	0.796	0.387–1.635	0.534	0.981	0.568–1.693	0.944						
Hepatic encephalopathy	22	1.334	0.602–2.957	0.478	1.728	0.939–3.182	0.079						
Hepatorenal syndrome	50	0.944	0.473–1.884	0.870	1.107	0.643–1.908	0.714						
Pretransplant ventilator care	22	1.551	0.720–3.341	0.262	1.974	1.099–3.548	0.023	2.004	0.904–4.442	0.087	1.723	0.900–3.298	0.101
Prehospitalization	91	2.106	0.503–8.806	0.308	1.532	0.611–3.844	0.363						
Pretransplant inotropics	32	1.126	0.548–2.314	0.747	1.237	0.697–2.195	0.468						
Simulect induction	58	0.601	0.286–1.263	0.179	0.990	0.557–1.760	0.973						
Mean daily FK trough level ≥ 9 ng/mL	9	3.692	1.288–10.587	0.015	2.879	1.251–6.625	0.013	2.935	1.195–7.211	0.019			
Mean daily MMF dosage ≥ 500 mg	34	1.009	0.454–2.240	0.983	1.002	0.555–1.807	0.996						
Male donor (vs. female)	64	1.145	0.566–2.316	0.706	1.123	0.647–1.948	0.680						

*LT* Liver transplantation, *reLT* Liver retransplantation, *RRT* Renal replacement therapy, *MELD* Model for end-stage liver disease, *GRWR* Graft-recipient weight ratio.

## Discussion

Although the outcome of LT is improving, some of the recipients experience graft failure which requires reLT. Unlike first LT which shows excellent outcome, reLT generally shows poor outcome in that the recipient is already immunocompromised due to the use of immunosuppressants, comorbid condition before transplantation and also due to the surgical difficulties made by adhesions after the first LT.

The main findings of this study are reconfirmation of the morbid outcome of reLT and the confirmation of the importance of GRWR and lowered immunosuppression in reLT recipients. Although the survival outcome varies among published studies, all the studies reported poor outcome compared to first LT. Our data showed relatively poor outcome compared to other literatures maybe due to poor patient condition at the time of reLT.

Our 25-year single center experience on reLT showed 5% (n = 121) of reLT among 2392 recipients. Other published study reported reLT rates ranging from 5 to 22%^2,3,6,9^. Acute graft dysfunction after prior LT comprised 42.7% (n = 44) of total reLT case while subacute or chronic graft dysfunction comprised 57.3% (n = 59). Primary nonfunction (n = 22, 21.4%) was diagnosed when the graft failed to function properly without evident vascular compromisation. Secondary graft nonfunction (n = 22, 21.4%) was diagnosed when the graft failed to function due to vascular complication or ischemic damage due to shock of any reason. In the study by Kuramitsu et al., indications for reLT were vascular (8.1%), small-for-size syndrome (1.6%) and unknown in 28.5%. Although data were imperfect due to the study’s limitation using national registry, rejection (26.0%), recurrence (29.3%) and bile duct complications (6.5%) comprised 61.8% of cases which was similar to our study^3^. A single center study published by Marudanayagam et al. reported similar data although primary nonfunction (10.7%), hepatic artery thrombosis (31.1%) and graft infarction (13.2%) comprised 55.0% which is a little higher than our data.

However, our study showed different demographics especially on how the reLT was performed. The median MELD score of our recipients was 34 (IQR 26–40) and 88.3% (n = 91) of the recipients received their graft from deceased donors. The study by Marudanayagam et al. reported only deceased donor liver transplantation and the multicenter study of Japan also showed living donor dominance (73.2%) during reLT. The median MELD score was 22.3 (range 6–40) in the study by Marudanayagam et al. and ranged 26 to 30 in the study by Mezochow et al., which reported 3483 adult reLT recipients from the United Network for Organ Sharing database^3–5^. These differences show that although the background history of reLT is similar, the clinical course differ between countries with different regulation and culture. Unfortunately, our data showed that LT recipients who require reLT do not have plenty of other option but to wait for allocation with a deceased donor of which the chance is relatively lower than other countries due to the shortage of organ donation from deceased donors. Consequently, patients who require reLT only be allocated after their conditions become more worse, which is represented by high RRT rate at the time of reLT (45.6%) compared to 17.3% in the study by Mezochow et al.^5^.

In this study, we reviewed the data on immunosuppression. The main induction regimen except for steroid is basiliximab in our institution. Except for two cases with antithymocyte globulin, basiliximab was used. Categorizing the use of induction regimen was difficult since certain proportion of patients underwent reLT in a short period from their prior LT. A total of 38 cases (36.9%) were performed without an induction regimen except for steroid. Among them, 18 cases were due to the short-term use of induction regimen from prior LT. This is in fact quite high when we compare it to the study by Mezochow et al. which reported 80.4% of no induction rate^5^. In the study, they reported that depleting induction marginally improved post-reLT mortality (HR 0.77, CI 0.61–0.99, P = 0.08) However, interpreting these data require caution in that the intensity of immunosuppression must have been adjusted for each patient based on their clinical condition. Although there was no statistical difference in the immunosuppression between reLT with partial liver graft and whole liver graft, the partial liver graft group showed higher rate of basiliximab induction. Furthermore, initiation date was earlier and mean trough level of tacrolimus was higher. The background characteristics of reLT group with partial liver graft generally shows better pretransplant condition compared to the whole liver group in regards of liver chemistries and pretransplant hospitalization. These data shows that intensity of immunossupression was adjusted for morbid patients. In the absence of direct parameter for the net state of immunosuppression, lowering the intensity of immunosuppression in reLT recipients seem to be a safe approach when combined with the finding that high FK trough level was related to poor outcome. Therefore, this study emphasizes the importance of lowering the level of immunosuppression in reLT patients who are already immunocompromised. Low immunosuppression can be a risk for rejection in LT. However, there was no difference in rejection rate between recipients with partial liver and whole liver.

GRWR showed conflicting outcomes in first LT recipients and reLT recipients. While higher GRWR being related to poor outcome in the univariable analyses of entire cohort, caution is required not to interpret the result without any consideration on selection bias. Low GRWR, which is related to living donor LT, are cautiously selected for adequate graft volume, therefore it is highly unlikely our data might have showed any evidence of low GRWR showing inferior outcome. In the previous study of our center, we showed that GRWR is not a significant factor related to the outcome in living donor LT^10^. On the other hand, the GRWR in relation to reLT cases showed opposite finding. Although GRWR failed to show statistical significance as a continuous variable in overall patient survival, GRWR less than 1% showed highly morbid outcome even after adjustment for the confounding variables. While GRWR of 1% generally is considered adequate for successful LT, the results devastating in reLT patients. The six patients with reLT using a graft with GRWR less than 1%, all expired after the transplantation. When the cutoff was elevated to 1.2%, 90.9% (10 out of 11 reLT cases) expired after reLT.

To avoid poor outcome in reLT, some strategies can be recommended. When the patient requires reLT, it is best to perform reLT when the patient is in their best condition. However, shortage of organ donation can limit this strategy, and living donor LT can be a solution in selected cases. This strategy can be implicated by the higher proportion of living donor LT in the Japanese national cohort study. However, deceased donor LT outweighted living donor LT in adult patients (61.0%) in the Japanese study, showing it can be only achieved in selected patients in contrast to pediatric reLTs. (16.2% deceased donor LT) The study by Kuramitsu et al. concluded that graft failure within a year should be thoroughly restricted to justify the use of living donors based on their result of worse outcome compared to graft failure exceeding 365 days^3^. The outcome was reproduced in the study of Mezochow et al., showing poor outcome in reLT timing between 91 to 365 days. (HR 1.41, CI 1.19–1.67) However, our study did not find a statistically significant outcome related to duration between LTs. Therefore, deciding whether to perform living donor LTs for patients who are available for their family members for living donation based on the timing of graft failure needs to be reconsidered and managed as an individualized approach. Given the information that reLT is related to worse outcome compared to first LT, building up a system to give advantage in deceased donor allocation can be a solution, while ethical issue for apportioning a public matter to a person who already received the benefit can be a conflicting issue. For preparing surgery for reLT patients, patient conditioning for the best operation can be needed since hypovolemic shock due to massive bleeding can compromise the allograft. The intensity of immunosuppression is also a key factor. A total of 15.5% (n = 16) of reLT recipients expired due to infection. Infectious condition prior to reLT can also influence graft failure. Although 11.5% of deaths were related to graft rejection after reLT, decreasing the intensity of immunosuppression in the early period can be justified since all four patients died for chronic rejection survived more than two years after reLT (median 44.1 months, range 25.5–88.9 months). Lowering the immunosuppression in the initial period and gradually elevating the intensity after patient stabilization can be a good strategy in the long term.

Other factors related to the outcome of LT were MELD score and donor age for primary graft survival, recipient age ≥ 60 years, hepatorenal syndrome, pretransplant albumin for overall patient survival. These factors are previously well-known factors related to the outcome^11,12^.

The limitation of this study is that it is a retrospective single-center study. However, regarding reLT, prospective trial is unrealistic. Although some studies based on multicenter registries have been published, the details in the data are lacking which can make the readers confused. One example is the study by Mezochow et al., showing marginal benefit of depleting induction regimen. Experienced transplant clinician would interpret the result cautiously by distinguishing the true cause and consequence while some readers might conclude that depleting regimen can be the key for better management. On the other hand, our study could collect the data in more detail, especially during the first month after reLT. We demonstrated that nearly half (n = 50, 48.5%) of the reLT recipients experienced tissue-invasive infection as well as viremia represented by cytomegalovirus antigenemia (n = 63, 61.2%). A comprehensive review of our reLT patient cohort made it possible to find clues in managing reLT recipients. Tacrolimus trough level which is a variable of post-transplant management must be interpreted with caution since the level itself is a post-transplant variable. Therefore, a separate analysis excluding post-transplant immunosuppression in Table 5. When excluded, the only factor related to poor survival was GRWR less than 1%. This emphasizes the importance of having enough liver graft volume to solve the metabolic demand of morbid recipient.

To summarize our data on reLT patients, it is important to acknowledge that reLT patients are at high risk of mortality after LT compared to first LT. For these specific group of patients, liver graft with plenty of GRWR should be chosen with minimizing immunosuppression in the initial period.

### Supplementary Information

Supplementary Information.

## Data Availability

The data related to this study can be provided to whom requests after achievement of approval by the corresponding author (Jong Man Kim) by requesting review for data availability to the institutional ethical review board of Samsung Medical Center.

## Abbreviations

LT: Liver transplantation
reLT: Liver transplantation
MELD: Model for end-stage liver disease
RRT: Renal replacement therapy
CMV: Cytomegalovirus
IQR: Interquartile range
CTP: Child-Turcot-Pugh
GRWR: Graft-recipient weight ratio
