# Supplementary material for: Graft-recipient-weight ratio and lowered immunosuppression is important for the success of adult liver retransplantation
Source: Sci Rep. 2023 Aug 7;13:12778. doi: 10.1038/s41598-023-39007-7 (PMC10406835; doi:10.1038/s41598-023-39007-7)
Supplement: Supplementary file 1 — Supplementary Information. [file 41598_2023_39007_MOESM1_ESM.docx]

Supplementary Table 1. Multivariable Cox analyses for potential risk factors for overall graft survival in patients who underwent liver transplantation.

| variables | n | Overall graft survival | | | | | |
| --- | --- | --- | --- | --- | --- | --- | --- |
|  |  | Univariable | | | Multivariable (final model) | | |
|  |  | HR | CI | P | HR | CI | P |
| Retransplantation | 103 | 3.788 | 2.900-4.949 | <0.001 | 3.026 | 2.233-4.101 | <0.001 |
| Partial graft (against whole) | 1659 | 0.634 | 0.538-0.747 | <0.001 |  |  |  |
| MELD score |  | 1.018 | 1.011-1.025 | <0.001 | 1.014 | 1.007-1.021 | <0.001 |
| Male recipient (vs. female) | 1635 | 0.981 | 0.822-1.172 | 0.836 |  |  |  |
| Recipient age≥60 | 509 | 1.291 | 1.075-1.550 | 0.006 | 1.294 | 1.066-1.570 | 0.009 |
| Hepatocellular carcinoma | 1139 | 1.077 | 0.923-1.257 | 0.346 |  |  |  |
| Living donor (vs. deceased donor) | 1633 | 0.633 | 0.537-0.745 | <0.001 |  |  |  |
| Hypertension | 277 | 1.135 | 0.895-1.439 | 0.295 |  |  |  |
| Diabetes mellitus | 481 | 1.120 | 0.932-1.348 | 0.227 |  |  |  |
| Hepatic encephalopathy | 522 | 1.065 | 0.896-1.267 | 0.473 |  |  |  |
| Variceal bleeding | 352 | 0.795 | 0.646-0.978 | 0.030 |  |  |  |
| Ascites | 1304 | 0.786 | 0.672-0.920 | 0.003 | 0.855 | 0.719-1.017 | 0.077 |
| Hepatorenal syndrome | 316 | 2.012 | 1.642-2.465 | <0.001 |  |  |  |
| Spontaneous bacterial peritonitis | 210 | 1.006 | 0.797-1.271 | 0.959 |  |  |  |
| Pretransplant ventilator care | 116 | 1.696 | 1.244-2.312 | 0.001 |  |  |  |
| Pretransplant albumin |  | 1.170 | 1.098-1.246 | <0.001 | 1.191 | 1.116-1.270 | <0.001 |
| ABO incompatible | 225 | 0.920 | 0.684-1.236 | 0.580 |  |  |  |
| Male donor (vs. female) | 1427 | 0.906 | 0.772-1.062 | 0.906 |  |  |  |
| Donor age |  | 1.016 | 1.011-1.021 | <0.001 | 1.011 | 1.005-1.017 | <0.001 |
| Warm ischemic time |  | 1.001 | 0.997-1.004 | 0.714 |  |  |  |
| Cold ischemic time |  | 1.001 | 1.001-1.002 | <0.001 |  |  |  |
| GRWR |  | 1.441 | 1.264-1.642 | <0.001 |  |  |  |
| GRWR<1.0 | 672 | 0.769 | 0.645-0.917 | 0.003 |  |  |  |

Supplementary Table 2. Multivariable Cox analyses for potential risk factors for overall graft survival in patients who underwent retransplantation.

| Variables | n | Overall graft survival | | | | | |
| --- | --- | --- | --- | --- | --- | --- | --- |
|  |  | Univariable | | | Multivariable (FK included) | | |
|  |  | HR | CI | P | HR | CI | P |
| GRWR |  | 0.769 | 0.504-1.172 | 0.222 |  |  |  |
| GRWR < 1.0% | 6 | 0.299 | 0.125-0.712 | 0.006 | 3.029 | 1.018-9.018 | 0.046 |
| Living donor for first transplant | 75 | 0.858 | 0.481-1.531 | 0.604 |  |  |  |
| Living donor for retransplant | 12 | 1.434 | 0.704-2.920 | 0.321 |  |  |  |
| Acute dysfunction (against chronic) | 44 | 0.903 | 0.538-1.515 | 0.699 |  |  |  |
| Days between transplantations ≥ 30 days | 70 | 1.553 | 0.865-2.788 | 0.141 |  |  |  |
| Partial graft (against whole) | 15 | 1.384 | 0.717-2.670 | 0.332 |  |  |  |
| MELD score |  | 1.008 | 0.978-1.039 | 0.607 |  |  |  |
| Male recipient (vs. female) | 71 | 0.936 | 0.535-1.635 | 0.815 |  |  |  |
| Recipient age ≥ 60 years | 19 | 1.107 | 0.584-2.098 | 0.755 |  |  |  |
| Hepatocellular carcinoma | 37 | 1.029 | 0.610-1.735 | 0.915 |  |  |  |
| Hepatic encephalopathy | 22 | 1.384 | 0.758-2.526 | 0.290 |  |  |  |
| Hepatorenal syndrome | 50 | 0.913 | 0.543-1.537 | 0.733 |  |  |  |
| Pretransplant ventilator care | 22 | 1.582 | 0.890-2.812 | 0.118 |  |  |  |
| Prehospitalization | 91 | 1.251 | 0.537-2.915 | 0.603 |  |  |  |
| Pretransplant inotropics | 32 | 1.049 | 0.603-1.825 | 0.866 |  |  |  |
| Simulect induction | 58 | 0.850 | 0.494-1.463 | 0.557 |  |  |  |
| Mean daily FK trough level ≥ 9 ng/mL | 9 | 2.793 | 1.277-6.107 | 0.010 | 2.716 | 1.201-6.141 | 0.016 |
| Mean daily MMF dosage ≥ 500mg | 34 | 0.969 | 0.549-1.711 | 0.914 |  |  |  |
| Male donor (vs. female) | 64 | 1.229 | 0.722-2.093 | 0.447 |  |  |  |
